# How Did the COVID‐19 Restrictions Change the Digital Contact of People With Intellectual Disabilities? A Longitudinal Multi‐Method Study in Sheltered Home Care Facilities

**DOI:** 10.1111/jar.70140

**Published:** 2025-11-02

**Authors:** Eline Wagemaker, Lianne Bakkum, Laura Tissing, Loïs van de Water, Noud Frielink, Petri J. C. M. Embregts, Ines van Keer, Annet ten Brug, Paula S. Sterkenburg, J. Clasien de Schipper, Anne Tharner, Carlo Schuengel

**Affiliations:** ^1^ Academische Werkplaats Viveon Vrije Universiteit Amsterdam Amsterdam the Netherlands; ^2^ Department of Clinical Child and Family Studies Vrije Universiteit Amsterdam Amsterdam the Netherlands; ^3^ 's Heeren Loo Amersfoort the Netherlands; ^4^ Netherlands Institute for Health Services Research (Nivel) Utrecht the Netherlands; ^5^ Academische Werkplaats Leven met een verstandelijke beperking, Tranzo, Tilburg School of Social and Behavioral Sciences Tilburg University Tilburg the Netherlands; ^6^ Faculty of Psychology and Educational Sciences, Research Unit Parenting and Special Education Catholic University of Leuven Leuven Belgium; ^7^ Academische Werkplaats EMB University of Groningen Groningen the Netherlands; ^8^ Academische Werkplaats Affect‐Us Vrije Universiteit Amsterdam Amsterdam the Netherlands; ^9^ Bartiméus Zeist the Netherlands

**Keywords:** COVID‐19, digital contact, intellectual disabilities, time trends

## Abstract

**Background:**

During the COVID‐19 pandemic, people with intellectual disabilities in sheltered home care facilities had to use digital contact as an alternative to face‐to‐face contact.

**Method:**

This study assessed digital and face‐to‐face contact and sentiment around digital contact among people with different levels of intellectual disability before, during and after the COVID‐19 restrictions (2016–2023) by analysing 575,348 daily records from a care organisation and yearly surveys from 342 relatives.

**Results:**

In the care records, mentions and positive sentiment around digital contact increased at the start of the restrictions and stabilised after the restrictions. According to relatives, digital contact remained similar over this period, while face‐to‐face contact was diminished during the restrictions.

**Conclusion:**

If the increased attention and predominantly positive sentiment to digital contact after the COVID‐19 restrictions in one large care organisation is indicative of a wider shift in long‐term care, more is needed for increasing contact with relatives.

**Summary**

- In the daily records of a care organisation for people with intellectual disability, digital contact was mentioned more frequently and more positively during and after the COVID‐19 restrictions than before.
- Yearly surveys filled out by family members of people with mild and moderate intellectual disability showed a temporary decrease in face‐to‐face contact during the COVID‐19 restrictions, while digital contact remained stable from 2016 to 2023.
- Digital contact offers a complementary form of social contact that could strengthen and broaden social networks of people living in sheltered home care facilities and thereby contribute to their digital inclusion and participation.
- To actually achieve more digital contact with relatives, increasing attention and positive sentiment among professional caregivers may not be sufficient.

## 1 Background

During the initial phase of the COVID‐19 pandemic, people with intellectual disabilities in sheltered home care facilities (approximately 70,000 in the Netherlands) faced visitation restrictions. For example, it was not allowed to receive visiting relatives or leave the care facility. For people with intellectual disabilities, who usually already have limited social networks (van Asselt‐Goverts et al. 2015), such restrictions could increase their loneliness. Digital communication, such as (video) calling, sending emails and text messaging, was used as an alternative to face‐to‐face interactions (Bakkum, Piekema, et al. 2023; Boeije et al. 2021; Chadwick et al. 2022; Nguyen et al. 2020). To gain insights into how the digital contact among people with intellectual disabilities changed as a result of the COVID‐19 restrictions, this study charted trends in digital contact in long‐term sheltered care homes.

Since the COVID‐19 pandemic, digital contact within home care facilities has received increased attention (Menschik et al. 2024). People with intellectual disability, relatives, volunteer visitors and direct support staff indicated using video calling, telephoning and text messaging, while key preconditions for digital contact, such as stable internet access and adequate support, needed improvement (Bakkum, Piekema, et al. 2023). Consequently, digital contact among people with intellectual disabilities may have been boosted during the pandemic, enhancing their digital participation and social inclusion (Chadwick et al. 2022). However, there are also indications that digital contact may have declined after the pandemic. For example, people with intellectual disabilities who lived in sheltered home care facilities, as well as their relatives, indicated that digital contact did not serve as a substitute for face‐to‐face contact but only as an addition (Douma et al. 2023). Additionally, some important barriers to digital contact may not have been addressed, such as sustained support for, in particular, people with severe disabilities (Bakkum, Piekema, et al. 2023). A lack of support after the COVID‐19 restrictions were lifted could cause digital contact to revert to baseline. A reduction in digital contact compared to the lockdown period could signal the potential negative impact of large‐scale but short‐term measures on digital inclusion.

Increases in digital contact of people with intellectual disabilities during the COVID‐19 pandemic do not necessarily mean that attitudes toward such contact are positive, especially if such digital contact was only present due to lack of other options for contact. Some people within sheltered home care facilities and their relatives reported positive changes in their stance (Bakkum, Piekema, et al. 2023; Chadwick et al. 2023; Douma et al. 2023), while others reported frustrations or described limited opportunities for meaningful social contact (Araten‐Bergman and Shpigelman 2021; Bakkum, Piekema, et al. 2023; Embregts et al. 2022). Thus, it remains unclear how positive and negative sentiments surrounding digital contact changed as a result of the COVID‐19 restrictions.

Most existing studies on digital contact among people with intellectual disabilities rely on cross‐sectional data (Bakkum et al. 2022; Bakkum, Piekema, et al. 2023; Chadwick et al. 2022; Douma et al. 2023). To understand how the rapidly changing environment during the COVID‐19 pandemic affected digital contact, it is important to examine potential long‐term changes across the periods before, during and after the pandemic restrictions. Another common limitation in prior research is the reliance on data from a single informant, typically a family member (e.g., Araten‐Bergman and Shpigelman 2021). Including multiple informants can provide a broader perspective on digital contact in this population. Furthermore, many studies have employed convenience sampling (e.g., Chadwick et al. 2023; Douma et al. 2023), which may limit both the generalisability across groups and the diversity of participants.

To address these limitations, the present study adopted a dual‐perspective longitudinal design, drawing on large‐scale data collected between 2016 and 2023, covering periods before, during and after COVID‐19 restrictions. This included: (a) daily professional caregiver reports extracted from clients' electronic care records (see Bakkum, Bisschops, et al. 2023; Schuengel et al. 2020; Zaagsma et al. 2020 for similar approaches) and (b) annual surveys completed by relatives of people with intellectual disabilities living in sheltered care homes. Both methods yield unique information, and combining them allows for more robust findings and more nuanced conclusions.

To get a first impression of changes in sentiments surrounding digital contact, the present study applied sentiment analysis to daily reports of professional caregivers, using a network‐based language model. This approach addresses several biases common in prior research using caregiver or client reported data (e.g., Araten‐Bergman and Shpigelman 2021; Bakkum, Piekema, et al. 2023; Chadwick et al. 2023; Douma et al. 2023). First, selection bias of the sample of people with intellectual disabilities is reduced because participation in the study does not place any burden on them. Second, retrospection bias, which arises when caregivers recall past changes, is reduced by relying on daily reports rather than retrospective accounts. Third, participant expectation bias, which occurs when responses are influenced by perceived researcher expectations, is mitigated because caregivers were unaware that their reports would be used for this research question.

The current study aims to answer three research questions. First, we investigated general time trends in (a) digital contact, (b) positive and negative sentiments around digital contact and (c) face‐to‐face contact. Second, we compared changes in these constructs over the periods before, during and after COVID‐19 restrictions. We hypothesized an increase in digital contact after the introduction of the COVID‐19 restrictions compared to the time before the restrictions. Third, we studied whether these trends were associated with clients' severity of intellectual disability and/or support needs. Based on previous research, we anticipated that people with lower levels of intellectual disability would engage in digital contact less frequently compared to people with higher levels of intellectual disability (Bakkum et al. 2022, Bakkum, Piekema, et al. 2023; Douma et al. 2023).

## 2 Method

### 2.1 Care Records

#### 2.1.1 Data Items

This study was conducted in collaboration with a large long‐term care facility for people with intellectual disabilities in the Netherlands. Clients or their legal representatives who had granted permission for the use of electronic care records for scientific research between 2016 and 2023 were contacted again with information about this specific study. They were given the opportunity to opt out within 2 weeks; however, none of the participants declined, resulting in an average sample size of 2481 unique participants (range 1968–2743) per year. This study was approved by the ethical review board of the Vrije Universiteit Amsterdam [removed for blinded review] and preregistered on OSF link.

We mined reports related to digital contact from the care records using a set of predefined Dutch search terms, which are fully listed in Table S1. These search terms were selected by three authors (E.W., L.B. and L.T.) and checked by the rest of the research team. Reports mentioning digital contact were aggregated weekly. These reports were labelled as either neutral, positive, or negative using the neural network‐based language model BERTje (de Vries et al. 2019). This model was first trained on 1000 manually coded reports, then cross‐validated, and we selected the best model based on confusion matrices. In Supporting Information A, more details about the text mining and sentiment analysis plan are included. With macro and weighted average scores all above 0.8, our model can be classified as good (see Table S2 and Figure S1; Sokolova and Lapalme 2009). The ratio of positive to negative reports about digital contact was calculated by first removing the neutral reports and then dividing the number of positive reports by the number of negative reports. A ratio of greater than one indicated more positive than negative reports.

We compared the time before the restrictions (before week 11, 2020) to the start of the restrictions (from week 11, 2020). Time was coded per week, from 1 for the first week to 416, as the final number of weeks from January 1, 2016, to December 31, 2023.

#### 2.1.2 Background Variables

We extracted the severity of intellectual disability from classifications in the clients' electronic care records, which were based on the DSM‐5 criteria (American Psychiatric Association 2013). Clients were categorized as having borderline intellectual functioning, mild, moderate, severe, or profound intellectual disability. Support needs (Dutch: *Zorgzwaartepakket* or *ZZP*) were also extracted from clients' electronic care records. This variable consisted of nine categories ranging from little support to very intensive residential support with full care and nursing. Three subgroups were created: support without behavioral regulation (ZZP1‐5), support with behavioral regulation (ZZP6, 7 and C) and support with full care and nursing (ZZP8).

#### 2.1.3 Procedures

The data manager of the care facility extracted care records from the database. The data scientist anonymised the care reports using the DEDUCE tool (Menger et al. 2018), conducted text mining, and trained, fine‐tuned and cross‐validated the Dutch natural language processing model (BERTje; de Vries et al. 2019). The weekly aggregated data was then shared with the researchers who performed the sentiment analysis. The sentiment analysis plan is summarized in Supporting Information A. The sentiment analysis procedure was developed by two researchers (E.W. and L.B.) in collaboration with the data scientist of the care facility (L.T.), partly following methods outlined in previous sentiment analysis research on Twitter (now called X; Rutte 2022).

#### 2.1.4 Data Analysis

Time‐series analyses were conducted using generalised linear modelling with a quasi‐Poisson distribution in R version 4.3.2 (R Core Team 2023) using R Studio (RStudio, 2023). First, the data were prepared and checked for irregularities using the *tidyverse* package (Wickham et al. 2019), then transformed into time series. The data were decomposed into seasonal and trend components, and seasonality was tested using the *seastests* package (Ollech 2019). If seasonality was detected, it was extracted from the data.

For the analyses with the counts of reports about digital contact as the outcome, generalized linear models with a quasi‐Poisson distribution were used. For the ratio of positive to negative sentiments regarding digital contact, linear regression models (ordinary least squares) were applied.

##### 2.1.4.1 Research Question 1: Time Trends in Digital Contact and Sentiments

Two separate sets of models were developed: one set with reports of digital social contact as the dependent variable, and one set with the ratio of positive to negative sentiments as the dependent variable. Time was included as an independent variable in both sets (Model 1).

##### 2.1.4.2 Research Question 2: Changes in Digital Contact and Sentiments Before, During and After the COVID‐19 Restrictions

The introduction of the COVID‐19 restrictions was added in both model sets as an independent variable (change in level; Model 2), and in an interaction term with time (change in slope; Model 3). Effect sizes for the level and slope change before and after the COVID‐19 restrictions were calculated using the *its2es* package (Travis‐Lumer 2024). For the counts of reports about digital contact, the effect sizes were presented as risk ratios (RR). For the ratio of positive to negative sentiments, effect sizes were calculated as Cohen's *d*.

##### 2.1.4.3 Research Question 3: Moderation by Severity of ID and Support Needs

All models were tested for each of the eight subgroups separately (borderline IF, mild, moderate, severe or profound intellectual disability, support without behavioural regulation, support with behavioural regulation, and support with full care and nursing). Goodness‐of‐fit between subsequent models was tested using Log‐likelihood ratio tests.

### 2.2 Panel Surveys

#### 2.2.1 Participants

Survey data were collected from relatives of people with mild or moderate intellectual disabilities, such as parents and siblings, who participated in a large, representative panel managed by the Netherlands Institute for Health Services Research (NIVEL, *Panel Living Together*). Recruitment was carried out through care facilities for people with intellectual disabilities, general practitioners, small‐scale care initiatives and snowball sampling throughout the Netherlands. Before enrolling in the study and upon receiving each survey, relatives were informed about the aim and procedure of the study, presentation of results derived from the data, and privacy regulations. The e‐mail address and telephone number of a fieldwork coordinator were provided. Relatives provided their preference for an online or paper‐and‐pencil survey and were encouraged to contact in case they desired further information. Participation was voluntary, and all relatives provided informed consent. The data were analysed anonymously and processed in accordance with the General Data Protection Regulation.

The panel started in 2016, initially consisting of 382 relatives who participated yearly. New relatives were added each year to maintain a stable response over time. On average, 71.8% (range 69.7%–74.3%) of the relatives responded to the survey invitations. For the current study, survey data were included according to the following criteria: (a) the person with intellectual disability lived in a sheltered home care facility (on average, 80.9% of respondents), (b) the person with intellectual disability did not live with their family, (c) the person with intellectual disability lived with other people with intellectual disabilities and (d) the person with intellectual disability lived in or near a supportive care organisation. If information on one of the criteria was missing, data for another criterion were checked. More inclusion information and a flow chart are included in Supporting Information B and Figure S2. The final sample consisted of 1265 timepoints from 2016 to 2023, spread across 342 unique relatives, who participated for an average of 3.7 years (SD = 2.35). The average sample size per year was 158 (SD = 18.1). Relatives were on average 66 years old (SD = 9.53) and included 58.3% females, 26.6% males and 4.1% other gender identities. Gender was missing for 10.9% of all relatives over the years. Descriptive statistics for the people with intellectual disabilities, as reported by their relatives, are shown in Table 1.

**TABLE 1:** Panel surveys: Descriptives of people with intellectual disabilities as reported by their relatives.

	2016	2017	2018	2019	2020	2021	2022	2023
*N*	193	166	174	150	143	141	152	146
% responses	74.3	71.8	73.7	71.6	70.7	69.7	70.4	72.0
Age in years								
*M*	49.4	47.8	49.8	51.1	51.2	49.4	46.7	48.3
SD	15.34	14.9	14.4	14.7	14.8	15.4	14.4	15.3
Range in years	23–89	23–83	24–84	26–83	26–84	19–85	19–86	20–87
% male	53.4	51.2	52.9	49.3	49	53.9	55.3	52.7
Severity of intellectual disability^a^								
% mild	38.9	40.4	40.8	36	42.7	35.5	33.6	34.9
% moderate	61.1	59.6	48.6	61.3	55.2	56	57.9	56.2

^a^ Severity of intellectual disability had the following numbers of missing values: 8 in 2018, 4 in 2019, 3 in 2020, 12 in 2021 and 13 in 2022 and 2023.

#### 2.2.2 Outcomes

##### 2.2.2.1 Face‐to‐Face Contact and Digital Contact

The surveys included one question about the frequency of face‐to‐face contact (i.e., ‘How often do you see your relative with intellectual disability?’) and one about the frequency of digital contact (i.e., ‘How often do you have contact with your relative with intellectual disability via telephone, SMS or social media (e.g., WhatsApp, Snapchat, or Messenger)?’). The questions were multiple‐choice with six answer options: every day, once or multiple times per week, once or multiple times per month, less than once per month, once or several times per year and almost never. In 2016 and 2017, the frequency of digital contact was measured in two separate questions for telephoning and text messaging. The highest frequency between the two was selected. Because the option ‘less than once per month’ was removed in 2022, we grouped this answer option with ‘once or several times per year’, resulting in five answer options. In 2022, the question about digital contact was replaced by a question on general contact (i.e., ‘How often do you have contact with your relative with intellectual disability?’), which did not provide sufficient data for describing digital contact. As a result, the digital contact data for 2022 were excluded.

##### 2.2.2.2 Background Variables

Relatives provided information about the age, gender and severity of intellectual disability of the person with intellectual disability, as well as their own age and gender. They also selected the living situation of the person with intellectual disability from four options (e.g., care institution, independent household) and the household partners of the person with intellectual disability from six options (e.g., with other people with intellectual disability, with family, alone). Both options slightly varied for the periods of 2016–2021 and 2022–2023. See Table S3 for the background variables per year.

##### 2.2.2.3 COVID‐19 Restrictions

We included the introduction of the COVID‐19 restrictions in the Netherlands (March 2020) as a categorical variable. We compared surveys before (2016–2019), during (2020–2021) and after the restrictions (2022–2023).

#### 2.2.3 Procedures

Relatives who were enrolled in the panel were contacted yearly in November by NIVEL via email and were asked to complete an online survey on various themes surrounding care and participation (Boeije et al. 2024). Completing the entire survey took approximately 30–40 min.

#### 2.2.4 Data Analysis

To answer our three research questions, we used cumulative link mixed models (R package ordinal; Christensen 2023), which are suitable for ordinal data that are nested within individuals. The data were analysed in R using RStudio (R Core Team 2023; RStudio 2023). We developed five hierarchical random‐intercept models for face‐to‐face and digital contact separately to examine time trends and changes in face‐to‐face and digital contact, as well as the moderation by severity of intellectual disability.

##### 2.2.4.1 Research Question 1: Time Trends in Face‐to‐Face and Digital Contact

The first model was an unconditional means model, containing only a random intercept at the participant level. In the second model, time (year of measurement, 2016–2023) was added as a fixed effect. In the subsequent models, we added polynomial transformations of time as fixed effects until these no longer significantly explained variance in the outcome variable.

Research question 2: Changes in face‐to‐face and digital contact before, during and after the COVID‐19 restrictions. In the next model, the COVID‐19 restrictions (before, during, after) were added as fixed effects.

##### 2.2.4.2 Research Question 3: Moderation by Severity of Intellectual Disability (Mild vs. Moderate)

In the last model, we examined whether the severity of intellectual disability (mild vs. moderate) moderated the trends in face‐to‐face and digital social contact before, during and after the COVID‐19 restrictions. To analyze improvements in model fit, Likelihood ratio model fit tests were used.

## 3 Results

### 3.1 Descriptive Statistics

#### 3.1.1 Care Records

A total of 575,347 unique care reports were extracted from the electronic database, each containing mentions of digital contact from 2016 to 2023. Table S4 provides additional details, including the weekly means, standard deviations and ranges for the total number of (positive and negative) reports, as well as for the ratio of positive to negative reports for both the total sample and subgroups. Out of the total data, 31 missing values were identified in the time series for the ratio of positive to negative sentiments because these weeks had zero negative reports (*n* = 28 in the profound intellectual disability group, *n* = 2 in the borderline intellectual functioning group and *n* = 1 in the support with full care and nursing group). These missing values were imputed using linear interpolation.

#### 3.1.2 Panel Surveys

**FIGURE 1:**
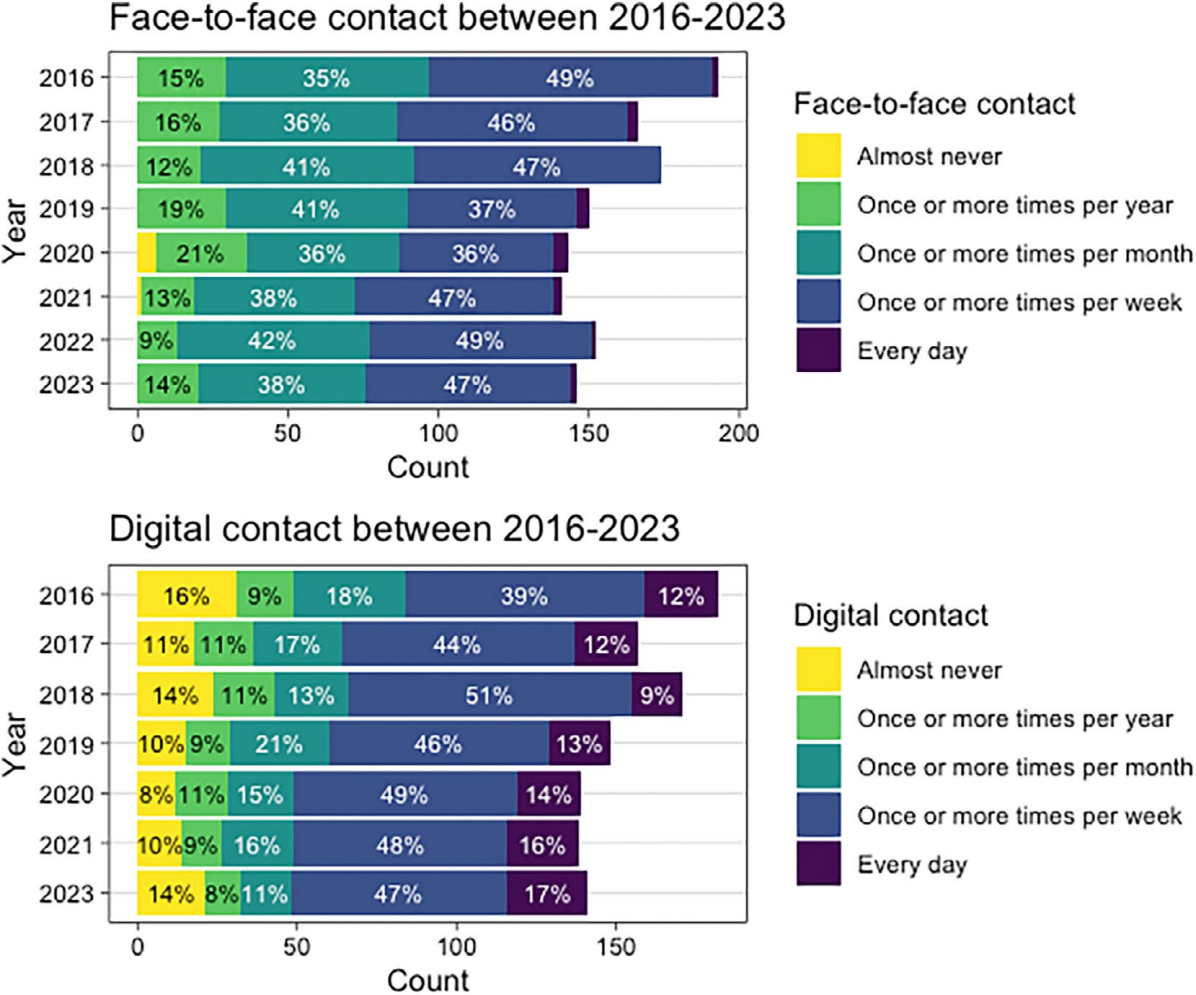
Percentages of the five ordinal categories of face‐to‐face contact and digital contact.

Figure 1 displays the frequencies (counts and percentages) of the five ordinal categories of face‐to‐face contact and digital contact from 2016 to 2023. Visual inspection of the figure suggests that both face‐to‐face contact and digital contact remained relatively stable over the years. The only notable change was a decrease in face‐to‐face contact during 2020.

### 3.2 Research Question 1: General Time Trends

#### 3.2.1 Digital Contact

**FIGURE 2:**
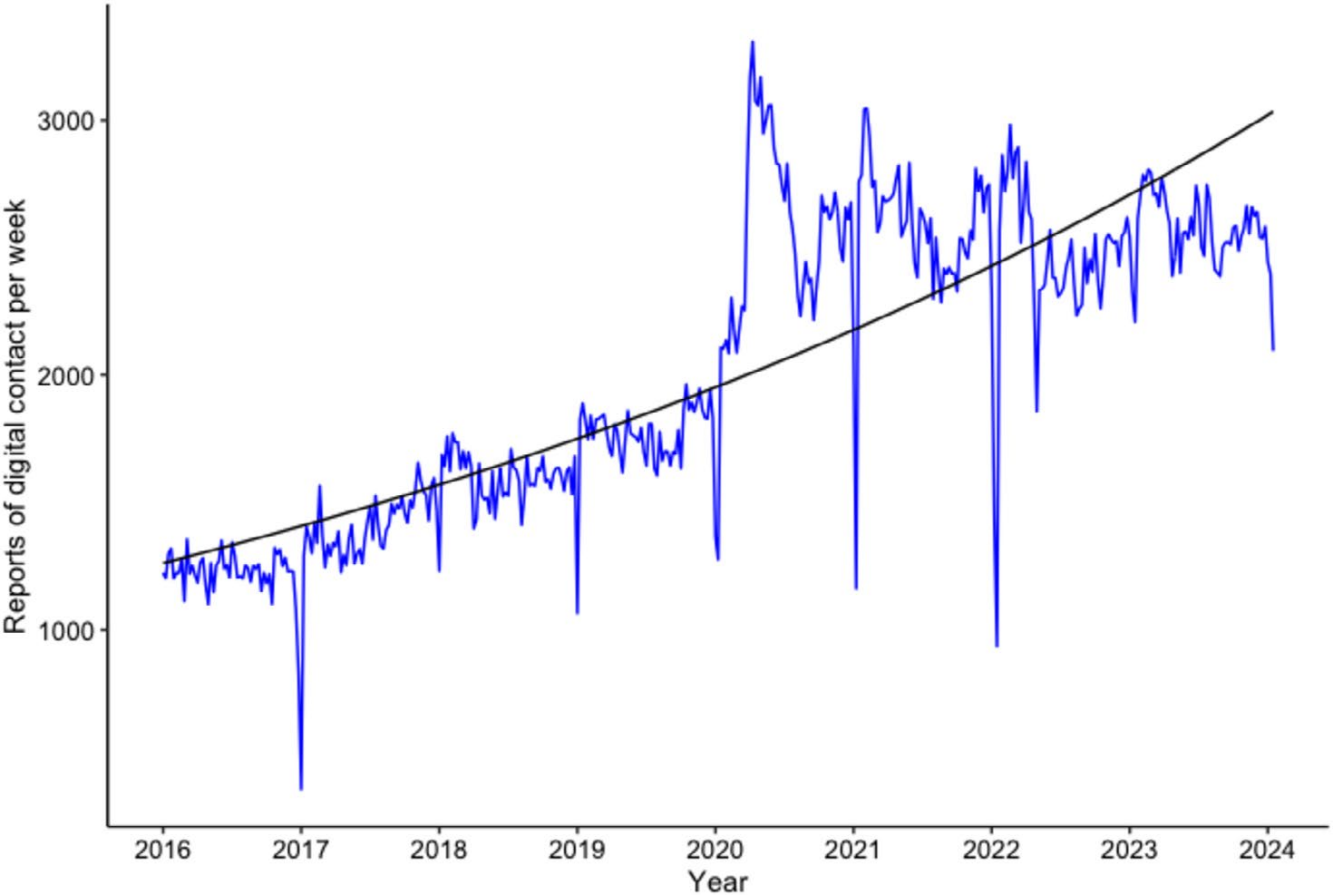
Care reports: Trend over time. The regression line represents the predicted values by the generalised linear quasi‐Poisson regression model (intercept + time).

In the care records, there was an increase over time in reports about digital contact (*b* = 0.002, SE = 0.00006, *p* < 0.001), as illustrated in Figure 2.

Results of the cumulative linked mixed models for digital contact, based on the panel survey data, are reported in Table 2. The linear trend over the years was non‐significant (*b* = 0.04, *p* = 0.436), nor was the quadratic transformation of time (*b* = −0.02, *p* = 0.165). Consequently, the quadratic transformation of year was dropped from the model.

**TABLE 2:** Results of panel survey data: Trends in digital contact and moderation by severity of intellectual disability.

	*b*	SE	∆DS
Model 1			
Intercept	36.83	6.07	
Model 2			7
Intercept	23.14	4.81	
Year	0.03	0.04	
Model 3			1.1
Intercept	23.17	4.81	
Year	0.23	0.15	
Year^2^	−0.02	0.02	
Model 4a			−8.7**
Intercept	36.58	6.0	
Year	0.07	0.09	
During vs. before COVID‐19 restrictions	0.06	0.32	
After vs. before COVID‐19 restrictions	−0.50	0.56	
Model 5			−3.34
Intercept	34.87	5.91	
Year	0.07	0.09	
During vs. before COVID‐19 restrictions	−0.25	0.40	
After vs. before COVID‐19 restrictions	0.38	0.67	
Level of ID	−2.02**	0.59	
During vs. before COVID‐19 restrictions × severity of intellectual disability	0.58	0.39	
After vs. before COVID‐19 restrictions × severity of intellectual disability	−1.34*	0.58	

*Note:* The intercepts represent the random effects (participant‐level). The reference category of COVID‐19 restrictions was: before the COVID‐19 restrictions (2016–2019). ∆DS = difference in deviance statistic (Log‐likelihood) compared to the previous model. *p < 0.05, **p < 0.01.

#### 3.2.2 Positive and Negative Sentiments Around Digital Contact

**FIGURE 3:**
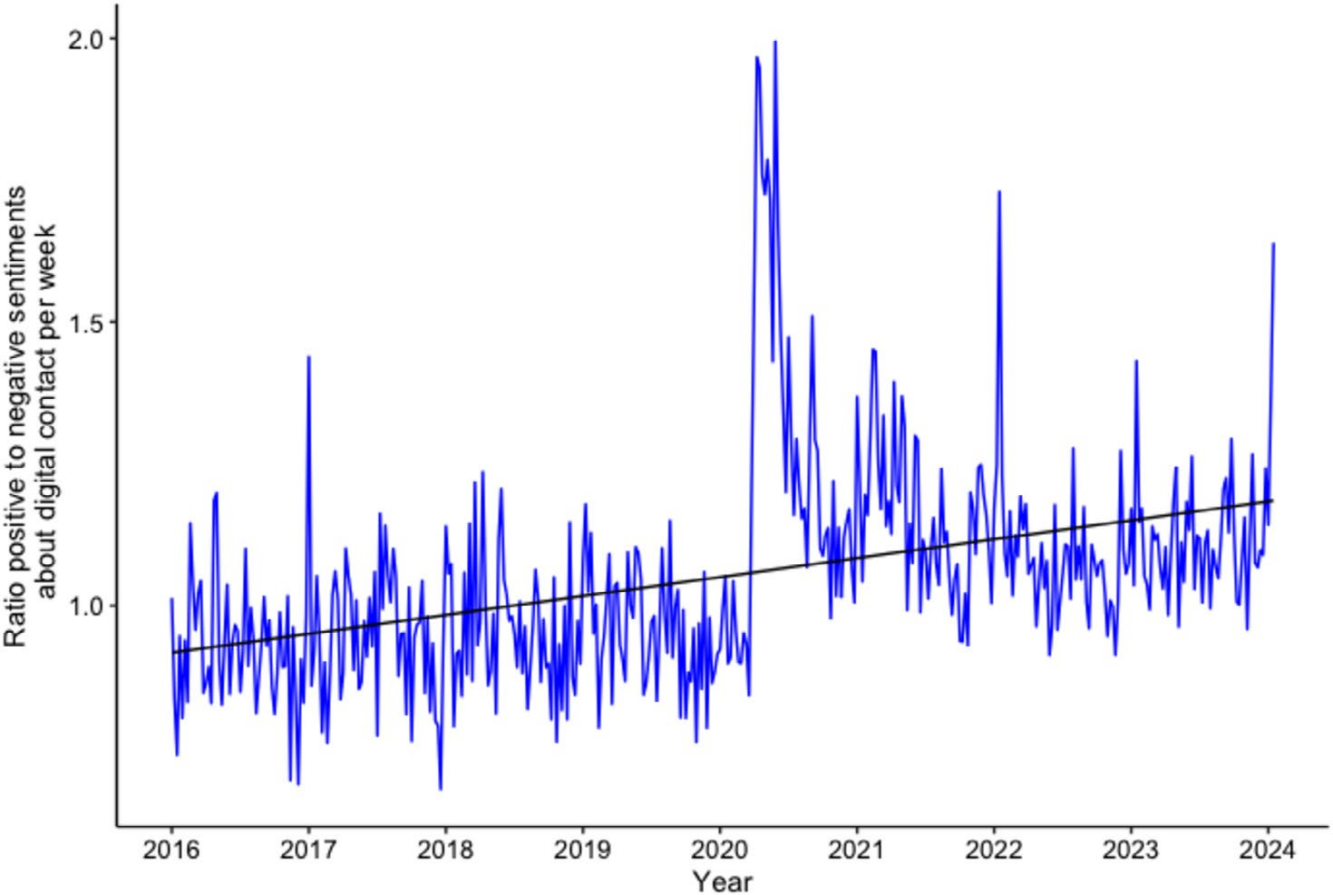
Care reports: Trend over time in the ratio of positive versus negative sentiments about digital contact. The regression line represents the predicted values by the generalized linear quasi‐Poisson regression model (intercept + time). A ratio > 1 indicates more positive than negative reports.

Additionally, the care records revealed a significant increase in positive sentiments about digital contact, reflected by an increase in the ratio of positive to negative sentiments (*b* = 0.0006, SE = 0.00007, *p* < 0.001; see Figure 3).

#### 3.2.3 Face‐to‐Face Contact

Results from cumulative linked mixed models for face‐to‐face contact, based on the panel survey data, are reported in Table 3. There was no significant linear trend (Model 2; *b* = −0.07, *p* = 0.090) or quadratic trend (Model 3; *b* = 0.02, *p* = 0.203) in face‐to‐face contact from 2016 to 2023. The quadratic time transformation was therefore dropped from subsequent models.

**TABLE 3:** Results of panel survey data: Trends in face‐to‐face contact and moderation by severity of intellectual disability.

	*b*	SE	∆DS
Model 1			
Intercept	24.34	4.93	
Model 2			−1.45
Intercept	24.7	4.97	
Year	−0.07	0.04	
Model 3			7.01
Intercept	18.95	4.35	
Year	−0.27	0.17	
Year^2^	0.02	0.02	
Model 4			4.07*
Intercept	19.22	4.38	
Year	0.01	0.09	
During vs. before COVID‐19 restrictions	−0.68*	0.34	
After vs. before COVID‐19 restrictions	−0.23	0.51	
Model 5			−3.34
Intercept	19.34	4.40	
Year	0.01	0.09	
During vs. before COVID‐19 restrictions	−0.86*	0.43	
After vs. before COVID‐19 restrictions	−0.96	0.58	
Severity of ID	−0.38	0.50	
During vs. before COVID‐19 restrictions × severity of intellectual disability	0.28	0.43	
After vs. before COVID‐19 restrictions × severity of intellectual disability	1.20*	0.47	

*Note:* The intercepts represent the random effects (participant‐level). The reference category of COVID‐19 restrictions was: before the COVID‐19 restrictions (2016–2019). ∆DS = difference in deviance statistic (Log‐likelihood) compared to the previous model. *p < 0.05.

### 3.3 Research Question 2: Changes Before, During and After the COVID‐19 Restrictions

#### 3.3.1 Digital Contact

**FIGURE 4:**
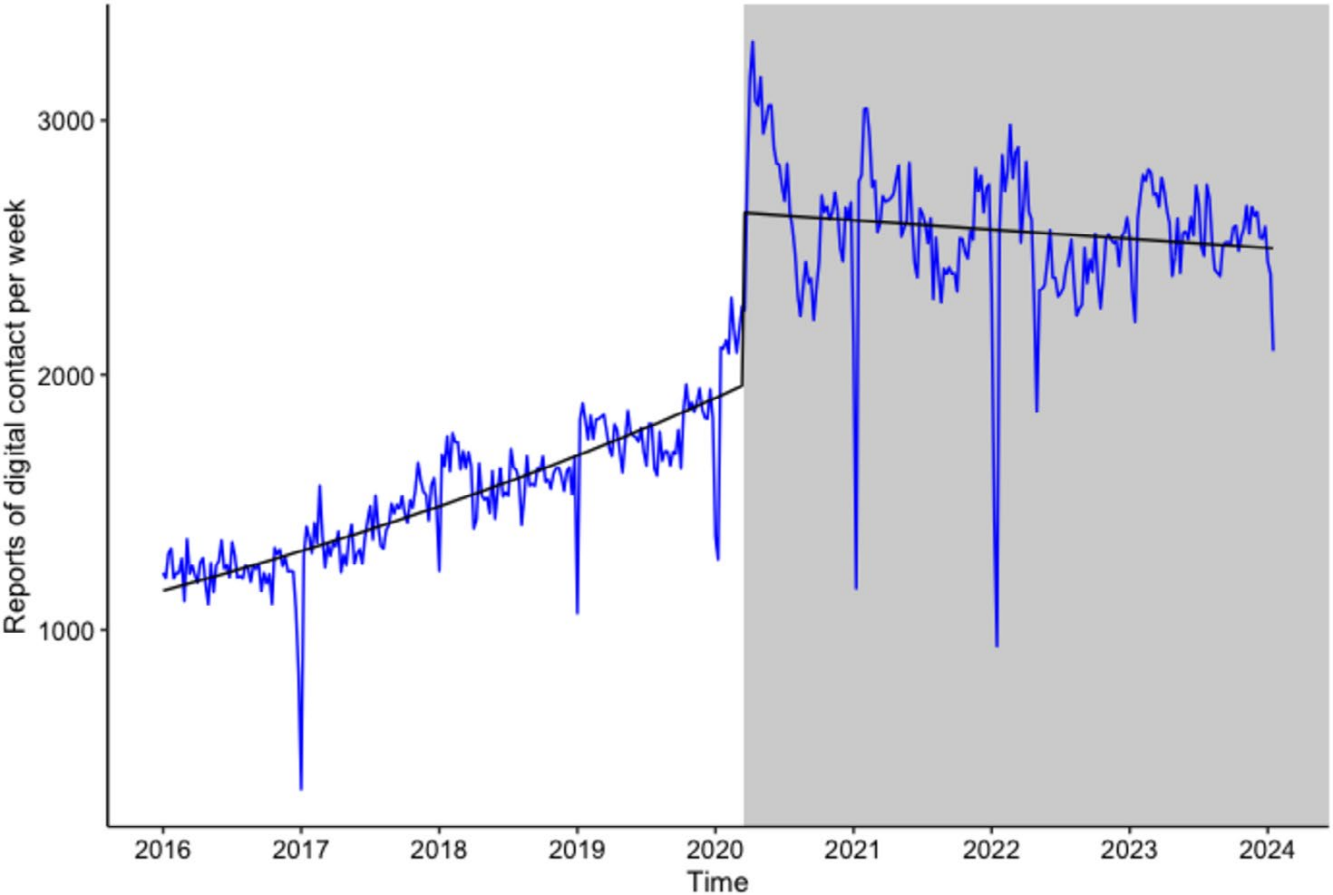
Care reports: Before and after the COVID‐19 restrictions trend over time. The regression line represents the predicted values by the generalised linear quasi‐Poisson regression model (intercept + time + start of COVID‐19 measures + time * start of COVID‐19 measures).

In the care records, there was a significant rise in reports about digital contact following the introduction of the COVID‐19 restrictions (change in level: *b* = 0.34, SE = 0.02, *p* < 0.001; see Figure 4). The number of reports increased by 40% after the pandemic restrictions (RR = 1.40, 95% CI [1.34; 1.47], *p* < 0.001). Additionally, a significant effect on the slope was observed (*b* = −0.003, SE = 0.0001, *p* < 0.001), indicating an increase in reports before the restrictions, followed by stability after (RR = 0.75, 95% CI [0.72; 0.78], *p* < 0.001).

In contrast, as shown in Table 2, we did not find significant differences in digital contact before versus during the COVID‐19 restrictions (*b* = 0.06, *p* = 0.848) and before versus after the restrictions (*b* = −0.49, *p* = 0.372) in the panel surveys. Additionally, we tested for the difference between during and after the pandemic (not reported in Table 2) and also found no significant effect (*b* = −0.56, *p* = 0.127).

#### 3.3.2 Positive and Negative Sentiments Around Digital Contact

**FIGURE 5:**
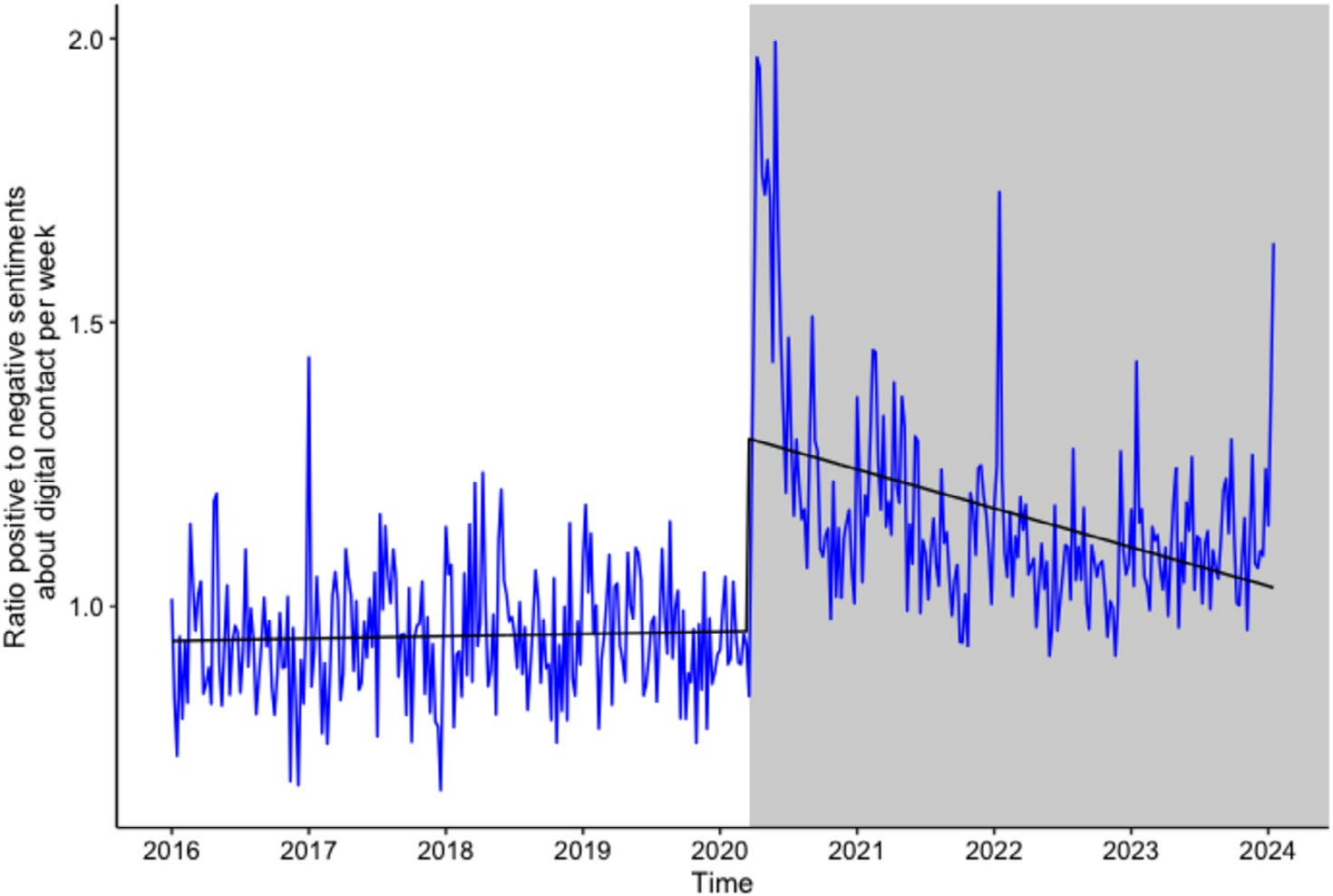
Care reports: Before and after the COVID‐19 restrictions trend over time in the ratio of positive versus negative sentiments about digital contact. The regression line represents the predicted values by the generalised linear quasi‐Poisson regression model (intercept + time + start of COVID‐19 measures + time * start of COVID‐19 measures). A ratio > 1 indicates more positive than negative reports.

For the care records, there was a significant increase in positive sentiments about digital contact after the COVID‐19 restrictions (change in level: *b* = 0.33, SE = 0.03, *p* <.001; see Figure 5). This was a large effect (Cohen's *d* = 4.70, 95% CI [3.24; 5.06], *p* < 0.001). The effect on the slope was also significant before versus after the restrictions (*b* = −0.001, SE = 0.0002, *p* < 0.001; Cohen's *d* = −1.42, 95% CI [−1.61; −1.08], *p* < 0.001).

#### 3.3.3 Face‐to‐Face Contact

As shown in Table 3, the panel reported significantly less face‐to‐face contact during the COVID‐19 restrictions compared to the period before (Model 4; *b* = −0.68, *p* < 0.05). However, no significant difference was found in face‐to‐face contact when comparing the periods before and after restrictions (*b* = −0.23, *p* = 0.646), nor was there a significant difference between during and after the COVID‐19 restrictions (*b* = 0.47, *p* = 0.139; not reported in Table 3).

### 3.4 Research Question 3: Moderation by Severity of Intellectual Disability and Support Needs

#### 3.4.1 Digital Contact

For the care records, we tested the same models used for research questions 1 and 2 separately for groups of clients with different levels of intellectual disability and support needs. The results are reported in Table 4. For most of the subgroups, the generalised linear quasi‐Poisson regression models (for the counts of digital contact reports) and the linear regression models (for the ratio of positive to negative sentiments) showed trends consistent with the total sample. Specifically, there was a significant positive trend over time (Model 1) in digital contact reports across all groups, indicating an increase in digital contact reports over the years from 2016 to 2023 (see Table 4). Additionally, for all groups, a significant effect of the COVID‐19 restrictions on both the intercept (level change; Model 2) and the slope of the trend over time in reports of digital contact (slope change; Model 3) was observed, reflecting similar patterns to the total sample. These results indicate a rise in reports about digital contact with the introduction of the COVID‐19 restrictions, followed by a stabilisation in the number of reports thereafter (i.e., flatter slope).

**TABLE 4:** Care reports: Results of generalised linear quasi‐Poisson regression models: Trends over time and effects of COVID‐19 restrictions per group.

Digital contact reports	Model 1: Trend over time		Model 2: Effect of COVID‐19 restrictions (level change)		Model 3: Time * Effect of COVID‐19 restrictions (slope change)	
	*B*	SE	*B*	SE	*B*	SE
Severity of intellectual disability						
Borderline intellectual functioning	0.003***	0.0001	0.34***	0.05	−0.007***	0.0003
Mild intellectual disability	0.002***	0.0001	0.23***	0.03	−0.003***	0.0002
Moderate intellectual disability	0.002***	0.0001	0.45***	0.02	−0.001***	0.0002
Severe intellectual disability	0.002***	0.0001	0.68***	0.03	−0.002***	0.0002
Profound intellectual disability	0.001***	0.0001	0.32***	0.04	−0.001***	0.0003
Support needs						
Support needs without behavioural regulation	0.003***	0.0001	0.25***	0.03	−0.003***	0.0002
Support needs with behavioural regulation	0.002***	0.0001	0.30***	0.02	−0.003***	0.0002
Support needs with full care and nursing	0.002***	0.0001	0.64***	0.03	−0.002	0.0003

Ratio of positive vs. negative sentiments	Model 1: Trend over time		Model 2: Effect of COVID‐19 restrictions (level change)		Model 3: Time * Effect of COVID‐19 restrictions (slope change)	
	*B*	SE	*B*	SE	*B*	SE
Severity of intellectual disability						
Borderline intellectual functioning	−0.0002	0.0002	0.16	0.08	0.0005	0.0007
Mild intellectual disability	0.0005***	0.00007	0.16***	0.03	−0.0005*	0.0003
Moderate intellectual disability	0.001***	0.0001	0.31***	0.06	−0.0003***	0.0004
Severe intellectual disability	0.002***	0.0004	0.83***	0.21	−0.005**	0.002
Profound intellectual disability	0.002*	0.001	1.16*	0.51	−0.006	0.004
Support needs						
Support needs without behavioural regulation	0.0003**	0.0001	0.35***	0.05	−0.00006	0.0004
Support needs with behavioural regulation	0.0006***	0.00007	0.31***	0.03	−0.001	0.0002***
Support needs with full care and nursing	0.0001*	0.0005	0.14	0.25	−0.003	0.002

*Note:* Effect of COVID‐19 restrictions was coded as 0 (before the restrictions started; reference category) or 1 (after the restrictions started). *p < 0.05, **p < 0.01, ***p < 0.0.

**FIGURE 6:**
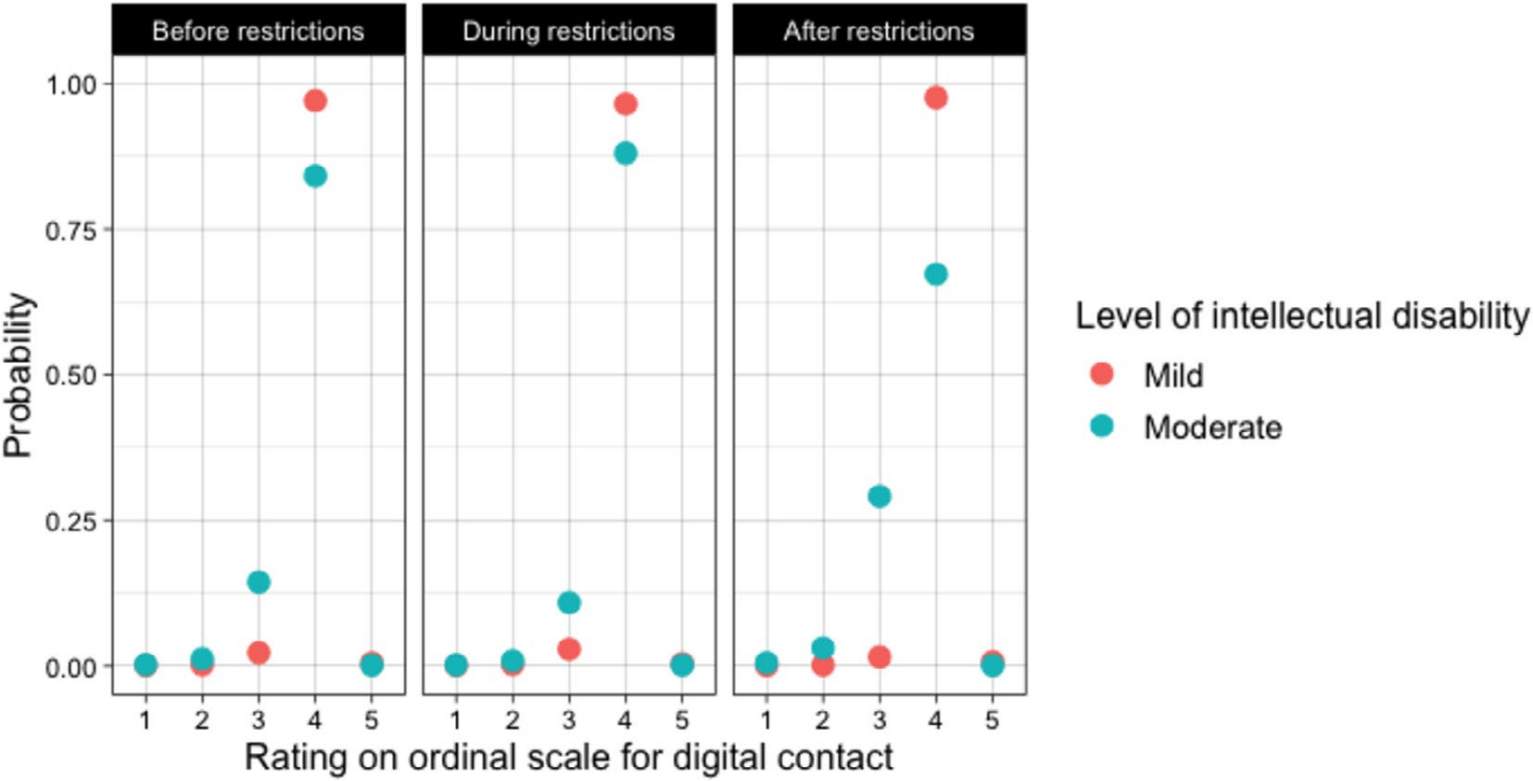
Panel surveys: Trends in digital contact before, during and after the COVID‐19 restrictions, Moderated by severity of intellectual disability. Ratings on the ordinal scale correspond to: (1) ‘Almost never’, (2) ‘Once or more times per year’, (3) ‘Once or more times per month’, (4) ‘Once or more times per week’, (5) ‘Every day’.

In the panel survey data, the interaction between severity of intellectual disability and the comparison of before versus during the COVID‐19 restrictions was non‐significant (*b* = 0.58, *p* = 0.135). However, the interaction between severity of intellectual disability and the comparison of before versus after the COVID‐19 restrictions was significant (*b* = −1.33, *p* < 0.05). As shown in Figure 6, after the COVID‐19 restrictions versus before, people with moderate intellectual disabilities had a higher probability of rating 3 on the ordinal scale (‘Once or more times per month’) and a lower probability of rating 4 on the ordinal scale (‘Once or more times per week’) compared to people with mild intellectual disabilities.

#### 3.4.2 Positive and Negative Sentiments Around Digital Contact

For the care records, there was a significant positive trend over time in positive sentiments about digital contact, for all groups based on the ratio of positive to negative sentiments (Model 1; Table 4), except for clients with borderline intellectual functioning. Furthermore, for most groups, the introduction of the COVID‐19 restrictions had a significant effect on both the intercept (level change; Model 2) and slope of the trend over time in positive sentiments about digital social contact (slope change; Model 3), except for clients with profound intellectual disability, borderline intellectual functioning, support needs without behavioural regulation and support with full care and housing.

##### 3.4.2.1 Post Hoc Analysis: Square‐Root Transformation of Time After Start of the COVID‐19 Restrictions

In Figures 4 and 5, we observed a non‐linear trend in time after the start of the COVID‐19 restrictions. To explore this further, we added a square‐root transformation of time to the part of Model 3 after the start of COVID‐19 restrictions for both outcomes (reports of digital contact and the ratio of positive/negative sentiments) as a post hoc analysis which was not pre‐registered. The results are mentioned in Supporting Information C and indicate a non‐linear association between time and both outcomes in the period after the COVID‐19 restrictions were implemented. Specifically, the effect of time on digital contact reports and sentiment ratios was strongest immediately after the introduction of the restrictions and gradually diminished over time.

#### 3.4.3 Face‐to‐Face Contact

In the panel survey data, the interaction between severity of intellectual disability and the comparison before versus during the COVID‐19 restrictions was non‐significant (*b* = 0.28, *p* = 0.510). However, there was a significant interaction between severity of intellectual disability and the comparison before versus after the COVID‐19 restrictions (*b* = 1.20, *p* < 0.05). Specifically, the difference between people with mild and moderate intellectual disabilities was larger after the COVID‐19 restrictions compared to before, as people with moderate intellectual disabilities had less digital contact after the COVID‐19 restrictions. Despite this, no significant improvement in model fit was observed after adding the interaction between severity of intellectual disabilities and the COVID‐19 restrictions.

## 4 Discussion

The present study examined trends in the frequency of digital contact and face‐to‐face contact of people with intellectual disabilities living in sheltered home care facilities, as well as positive or negative sentiment toward digital contact, from 2016 to 2023. First, we assessed general time trends in digital contact, sentiment regarding digital contact and face‐to‐face contact. Care records from 2016 and 2023 indicated an increase in the number of reports about digital contact, accompanied by a rise in positive sentiment toward such contact. In contrast, panel surveys revealed no time trends in either digital contact or face‐to‐face contact, suggesting that the frequency of both remained relatively stable between 2016 and 2023. Second, we examined changes before, during and after the COVID‐19 restrictions. Care records indicated a notable increase in documented digital contact during and after the restrictions compared to before the restrictions. In the panel surveys, the frequency of digital contact did not increase but remained stable, while the frequency of face‐to‐face contact declined during the COVID‐19 restrictions compared to before. Third, we examined whether these trends were associated with the severity of ID and/or support needs. Care records showed that the severity of intellectual disability and support needs were not associated with documented digital contact and sentiment over time. However, panel surveys demonstrated some differences between people with mild and moderate intellectual disabilities: people with moderate intellectual disabilities had less digital contact than people with mild intellectual disabilities, while people with mild intellectual disabilities had less face‐to‐face contact than people with moderate intellectual disabilities.

Our analyses of the time trends in digital contact and face‐to‐face contact from 2016 to 2023 further deepen understanding from earlier research, which mainly focused on the years during the COVID‐19 restrictions (Araten‐Bergman and Shpigelman 2021; Bakkum, Piekema, et al. 2023; Chadwick et al. 2022; Douma et al. 2023; Menschik et al. 2024). The findings of the present study reveal distinct patterns in digital and face‐to‐face contact across the periods before, during and after COVID‐19 restrictions. Contrary to our hypothesis, digital contact did not increase during the pandemic in the panel data, nor were differences observed before and after the restrictions. This lack of change might reflect challenges in accessibility or adaptation to digital platforms among the target population. This is consistent with findings by Chadwick et al. (2022), who highlighted significant barriers such as digital poverty, limited literacy and insufficient support as key contributors to digital exclusion for people with intellectual disabilities. In contrast, care records indicated a 40% increase in documented digital contact after the restrictions compared to before, suggesting a potential divergence between reports by relatives and caregivers. A potential explanation could be that caregivers reported daily on all digital contact of the client, while relatives only reported once a year limited to their own digital contact with their family member. Consequently, the reports from relatives might provide an underestimation of digital contact. For care practice, this underscores the value of using multiple informants and systematic reports to assess digital contact. Face‐to‐face contact declined during the restrictions compared to before, aligning with the anticipated impact of public health measures limiting physical gatherings (Lunsky et al. 2022). However, no differences were observed in face‐to‐face contact between the periods before and after the restrictions, suggesting a return to pre‐pandemic levels once restrictions were lifted.

With regard to sentiment, care records show a rise in positive sentiment associated with digital contact, suggesting rapid adaptation and acceptance of digital tools in response to the sudden shift in circumstances. For individuals reliant on care, digital contact may have offered a vital means of preserving social interaction during a challenging time. However, the observed stabilisation of both the reporting and sentiment after the initial surge suggests a nuanced, non‐linear pattern of adoption. This could reflect the normalisation of digital contact over time, where it transitioned from being a novel solution to becoming an integrated aspect of daily care routines. Also, early optimism about digital interaction might have given way to a more balanced perspective as users adapted to its limitations and benefits. For example, Chadwick et al. (2022) noted that while digital solutions like videoconferencing could mitigate social isolation, their effectiveness depended heavily on prior digital experience, adequate resources, digital support, as well as living conditions (group homes vs. living individually in an apartment)—factors that may have contributed to the contrasting patterns observed in our study.

Our findings also highlight differences in digital and face‐to‐face contact trends associated with the severity of intellectual disability. Based on the care records, findings indicate that the trends observed in the total sample regarding digital contact and sentiment were generally consistent across individuals with different levels of intellectual disability and support needs. However, for the subgroups at either end of the intellectual functioning and support needs continuum, changes in positive sentiment ratios were less pronounced or absent. Based on the panel surveys, people with moderate intellectual disabilities engaged less frequently in digital contact after the restrictions than people with mild intellectual disabilities, a trend that aligns with challenges such as limited digital literacy and insufficient support (Chadwick et al. 2022). Furthermore, they may live in group homes more often, which already provide face‐to‐face contact. Conversely, the stability of digital contact among people with mild intellectual disabilities could reflect greater familiarity with digital tools and higher digital support, as well as a higher need for social contact due to more isolated living conditions, such as living in a private apartment. For face‐to‐face contact, people with mild intellectual disabilities reported significantly less interaction after the COVID‐19 restrictions compared to those with moderate intellectual disabilities, indicating a prolonged negative impact on social engagement. Caton et al. (2024) found that social connections were a major motivator for internet use, particularly for people with strong family or support networks. This could help explain why individuals with mild intellectual disabilities, who may have more accessible or effective support, were able to adapt more easily to digital forms of connection compared to those with moderate intellectual disabilities, who might need sustained support to continue using digital contact.

## 5 Strengths and Limitations

The two most important strengths of this study were (a) access to chart trends through prospective longitudinal datasets that started long before the COVID‐19 pandemic, and (b) the dual perspectives from care reports and panel data of relatives. The number of care reports and the response rate of the panel study were high, with a considerable range of people with intellectual disabilities across age, gender, severity of intellectual disability and support needs. However, the two datasets had different setups regarding design, participating groups of people with intellectual disabilities, and measurement timepoints. This resulted in differing answers across datasets for each research question. The care records study included data from persons with borderline intellectual functioning to severe and profound intellectual disabilities, while the panel study provided data from relatives of people with mild or moderate intellectual disabilities. Compared to the yearly reports of the panel study, the weekly reports from the care records provided more granular insights into changes in the use of digital contact and sentiment.

We acknowledge four limitations of our study. First, both datasets on digital contact for people with intellectual disabilities may not have fully captured the construct. In the care reports, we relied on predefined search terms and automated classification of sentiment, which may have left out specific and rare sentiments. Also, from the care records, we could not identify whether digital contact was used by the person with intellectual disabilities, was between a relative and support staff, or among support staff. Relatedly, the positive or negative sentiment surrounding digital contact in the care records reflect the sentiment in the reports of support staff, which may derive only partially how persons with intellectual disability and relatives have experienced these contacts. Therefore, our care report results indicate the frequency of digital contact and the sentiment surrounding it within the care institution. Nevertheless, the panel data was limited to the digital contact between the relative and the person with intellectual disability, while the person with intellectual disability might have been engaging more in digital contact with others.

Second, our results may not generalise to all people with intellectual disabilities. Although the care records include a broader range of disability severities than the panel surveys, relatives of individuals with severe or profound intellectual disability were not represented in the survey and thereby input for this group is lacking. Also, although our study focused on people living in long‐term care facilities, the panel survey questions did not provide sufficient information to exclude people with intellectual disabilities who only received ambulatory care on the care facility's terrain. This group may have been less able to receive support to engage in digital contact compared to people who live in group homes. Nevertheless, our results show that digital contact remained relatively stable over time, especially for people with mild intellectual disabilities who most often receive ambulatory care.

Third, we did not take into account how factors such as digital literacy and access to technology may have affected digital contact, while we know from earlier work that these conditions vary across people and care settings (Bakkum, Piekema, et al. 2023). For the care reports, we assume that we recruited a diverse participant pool based on consent for the use of staff reports. In contrast, the panel data were primarily provided by parents of people with intellectual disabilities who were able to complete either online or paper‐and‐pencil surveys. Although none of these factors were measured, the relatives who were able and willing to fill in the yearly panel surveys might have had a relatively high socioeconomic status compared to other relatives of people with intellectual disabilities. Related to socioeconomic status, the participating relatives and their family members' access to (assistive) technology might have been better to some degree. Consequently, it is possible for the panel data to have presented a slightly optimistic view of digital contact between people with intellectual disabilities and their relatives.

Fourth, we did not distinguish between different types of digital communication tools used, but earlier work has provided insight into the broad range of tools used by people with an intellectual disability in the Netherlands (Bakkum, Piekema, et al. 2023). Future research may track the use of these tools over time to better understand how these meet their needs.

## 6 Conclusions and Implications

Text mining of care records revealed strong increases in both the number of mentions of digital contact and positive sentiment during the COVID‐19 visitation restrictions, followed by a decline after these restrictions were lifted, though levels remained higher than before the pandemic. In contrast, longitudinal surveys from family members showed a more stable picture: apart from a temporary drop in face‐to‐face contact during the restrictions, levels of both digital and face‐to‐face contact remained largely unchanged from 2016 to 2023. The persistence of positive sentiment beyond the period of restricted in‐person contact suggests a possible upward shift in the baseline for digital contact, although negative sentiment was also expressed. A direction for further research would be to investigate the extent to which such negative sentiment reflects practical difficulties, wishes and preferences of people with intellectual disabilities, or attitudes of support staff that may limit participation, such as tendencies toward overprotection (Hanzen et al. 2021; Nijs and Maes 2021).

These findings highlight both the potential and the challenges of sustaining digital contact for people with intellectual disabilities. Barriers such as digital poverty, limited literacy and insufficient support may continue to constrain participation, raising the question of whether efforts to build skills and improve support will be sufficient to close the widening digital divide since the pandemic (Murphy et al. 2024). People with intellectual disabilities may have a personal stake in engaging in digital contact as a means of fostering social inclusion (Caton et al. 2024), whereas exclusion from such contact can negatively impact their mental health (Baumgartner and Burns 2014).

The present study indicates that conditions for digital inclusion can be improved, even if this required pandemic emergency measures that led all to see the urgency of restoring some form of contact with loved ones. Clinicians are encouraged to promote digital contact for individuals with intellectual disabilities by facilitating access, tailoring technology to individual needs, ensuring digital safety and engaging in cross‐disciplinary collaboration to enhance well‐being and social participation (Murphy et al. 2024). Policymakers should strengthen the support structures required for digital inclusion beyond the pandemic, for example, by investing in digital skills, training caregivers in teaching digital skills, ensuring accessible communication and embedding co‐creation (Douma et al. 2023; Murphy et al. 2024). Given the ongoing digital transformation affecting all domains of life, support and facilities for digital participation should be integrated into standard care (Douma et al. 2023).

The increased attention and positive sentiment within care suggest that digital contact by people with intellectual disabilities living in sheltered home care facilities may become a normalised and expected part of care and support. Previous studies suggest that digital contact is not regarded as a substitute for in‐person contact (Araten‐Bergman and Shpigelman 2021; Bakkum, Piekema, et al. 2023), but it might offer a complementary form of social contact for people with intellectual disabilities that could strengthen and broaden their social networks (Lieberman and Schroeder 2020; Spassiani et al. 2023) and contribute to digital inclusion and participation (Hanzen et al. 2017). In line with recommendations from previous studies (Bakkum et al. 2022; Douma et al. 2023; Murphy et al. 2024), sustained support for digital contact may be needed before digital contact's potential to increase social participation of people with intellectual disability is finally realised.

## Author Contributions

All authors have made substantial contributions to the study and approved of the final version to be submitted. Lianne Bakkum, Noud Frielink, Petri J. C. M. Embregts, Annet ten Brug, Paula S. Sterkenburg, J. Clasien de Schipper, Anne Tharner and Carlo Schuengel contributed to the design of the study. Eline Wagemaker, Lianne Bakkum, Laura Tissing and Loïs van de Water contributed to data collection and analysis. All authors contributed to the writing of the article.

## Ethics Statement

The Ethics Review Board of the Vrije Universiteit (VCWE) approved the study protocol.

## Conflicts of Interest

The authors declare no conflicts of interest.

## Supporting information


**Data S1:** jar70140‐sup‐0001‐Supinfo.docx.

## Data Availability

The data that support the findings of this study are available on request in Yoda at 10.48338/VU01‐O2RBXW and 10.48338/VU01‐9C95LY. The metadata describing the data are available in Cedar at https://doi.org/10.60745/4932‐j592 and https://doi.org/10.60745/z0gk‐mm67.
