# Supplementary material for: How Did the COVID‐19 Restrictions Change the Digital Contact of People With Intellectual Disabilities? A Longitudinal Multi‐Method Study in Sheltered Home Care Facilities
Source: J Appl Res Intellect Disabil. 2025 Nov 2;38(6):e70140. doi: 10.1111/jar.70140 (PMC12580044; doi:10.1111/jar.70140)
Supplement: Supplementary file 1 — Data S1: jar70140‐sup‐0001‐Supinfo.docx. [file JAR-38-e70140-s001.docx]

**Supporting Information A: Care reports**

**Text mining and sentiment analysis plan**

1. **Selection of relevant folders**

We selected several subfolders of the electronic care records that may contain mentions of digital social contact. We selected: care plan/multidisciplinary consultation (i.e., practical support, annual plan discussion, and life history), goals (i.e., personal development, social inclusion, and other/particularities), agreements (i.e., devices, involvement of network/legal representative, social media and the internet, and remaining agreements), and annual evaluation with client (i.e., family and friends, house, and social inclusion).

1. **Importing the dataset**

The data manager of the care facility created a dataset in which each row represented a care report and added a week number (e.g., week 34 of 2020 became 202034) and imported 9,400,254 reports.

1. **Text mining**

To determine which care reports contained mentions of digital social contact, the researchers and the data scientist created search terms such as ‘calling’ or ‘WhatsApp’ (see Table S2). The data scientist of the care facility cleaned the data (i.e., remove double spaces, punctuation marks, and capitals) and filtered the dataset on reports that contained unique mentions of digital social contact (*N* = 575,348). The other reports were removed (N = 8,824,906).

**Table S1**

*Care Reports: Dutch Search Terms for Text Mining*

| **Type of digital contact** | **Dutch search terms** |
| --- | --- |
| Standard phone calling | ‘bellen’, ‘belt’, ‘belde’, ‘gebeld’, ‘telefoontje’, ‘belletje’, ‘telefonisch’, ‘telefoon’, ‘telefoneren’ ‘mobiel’, ‘mobieltje’ |
| Video calling | ‘videobellen’,‘beeldbellen’, ‘videocall’, ‘Zoom’, ‘Zoomen’, ‘Zoomde’, ‘Gezoomd’, ‘Teams’, ‘Facetime’, ‘Google Meet’, ‘Skype’, ‘schermcontact’ |
| Text messaging | ‘bericht sturen’, ‘berichtje’, ‘Whatsapp’, ‘appen’, ‘geappt’, ‘appte’, ‘appje’, ‘sms’, ‘smsen’, ‘iMessage’, ‘Signal’, ‘Telegram’, ‘chatten’, ‘texten’, ‘texting’, ‘voice bericht’, ‘audiobericht’, ‘voice app’ |
| Email | ‘email’, ‘mail’, ‘mailde’, ‘gemaild’, ‘Hotmail’, ‘Gmail’, ‘Outlook’ |
| Social media | ‘social media’, ‘Facebook’, ‘Messenger’, ‘Instagram’, ‘Insta’, ‘Twitter’, ‘TikTok’, ‘Snapchat’, ‘Forum’ |

1. **Anonymization**

The data scientist anonymized all care reports containing mentions of digital social contact using DEDUCE (Menger et al., 2018).

1. **Manual labelling of the data**

To train the artificial language model, the researchers manually labelled 1000 care reports as positive, negative or neutral (in line with a master thesis using a similar approach; Rutte, 2022). First, 200 reports were double coded by EW and LB, who reached a Cohen’s Kappa of .73. Second, potential differences in the coding were discussed and resolved. Third, the researchers independently coded the other 800 care reports. From these 1000 reports, 582 were coded as neutral (58,2%), 215 as positive (21,5%), and 203 (20,3%) as negative,

1. **Splitting the data**

The data scientist set apart 10% of the manually labelled data as test data. The remaining 90% was split into a training set (89%) and a validation set (11%), to obtain 100 reports for both the test and validation set. All sets were stratified to contain a similar distribution of neutrally, negatively, and positively coded reports. We selected a relatively large training set as the BERTje model has a preference for many training samples and used the “train_test_split” function from scikit-learn to perform the splitting.

1. **Training the model**

We selected the neural network-based model BERTje (de Vries et al., 2019). We trained the model using 81 combinations of the following selection of four hyperparameters:

1. Batch sizes of 4, 8, and 16 (Rutte, 2022). The batch size refers to the number of training reports that were used to update the model. A smaller batch size can lead to a more precise estimation of the gradient, but can also create more noise during the training processes. A larger batch size has the advantage of a higher learning pace, but also risks getting stuck in local minima instead of finding the global minimum.
2. Weight decays of .1, .01 en .0001 (Rutte, 2022). Weight decays add a penalty term to the loss function during training of the model. This penalty is proportional to the square of the model’s weights. It encourages smaller weight values, promoting simpler models and preventing overfitting. Thus, it strikes a balance between fitting the training data closely and maintaining the model’s ability to generalize to unseen instances.
3. Learning rates of .0002, .0003, and .0005. The learning rate determines how much the model’s parameters change in response to the calculated loss function. It metaphorically represents the speed at which the model “learns.” Choosing an appropriate learning rate involves a trade-off between convergence speed and overshooting. A too high learning rate may cause the model to jump over minima, while a too low rate leads to slow convergence or getting stuck in undesirable local minima.
4. Momentums of .85. 09, and .95. The momentum accelerates the optimization process during training by considering previous weight updates. It helps overcome limitations of standard gradient descent methods, such as slow convergence and oscillations around local minima. The current weight update is influenced by a percentage of the prior update vector, allowing the optimizer to continue advancing in the same direction as before.

After an epoch of each combination of parameters reached the lowest validation loss, five more epochs were allowed to check whether the validation loss could decrease further. The model with the lowest validation loss was saved.

1. **Cross-validating the model**

The performance of the model when using the different batch sizes (4, 8, 16) in combination with the 27 parameter combinations was compared with a train, validation and test set. The model was trained and validated, after which it was examined how the models performed on the test set. The evaluation was based on both a classification report and a confusion matrix. This showed that a batch size of 8 gave the best results on the test set, after which these models were selected for cross-validation. We applied 5-fold cross-validation to these 27 models.

1. **Selecting the best model based on confusion matrices**

Based on confusion matrices, we selected the hyperparameters with the best performance. As our analysis focuses on positive and negative sentiment, we had a preference for models that were good at distinguishing these categories from another. We selected a model with a weight decay of .1, a learning rate of .00002 and a momentum of .95.

1. **Testing the best model on the testing dataset**

We tested the model on the training data (as splitted in step 6). The confusion matrix results are shown in Table S3 and Figure S2. We checked the precision, recall, and f1-score for positive, neutral, and negative statements separately, as well as for the macro average and weighted average. Precision denotes the proportion of true positive predictions out of all positive predictions (i.e., specificity). Recall denotes the proportion of actual positive cases that were correctly identified by the model (i.e., sensitivity). The F1-score is a harmonic mean of precision and recall. Macro average emphasizes balanced performance across all labels, with special attention to rarer labels. Weighted average emphasizes overall performance, with more common labels contributing more to the final score. We specifically valued the macro and weighted average, as the distribution of neutral, positive, and negative statements in the care reports was unequal.

**Table S2**

*Care reports: Confusion Matrix Results*

|  | **Precision** | **Recall** | **F1-score** | **Support** |
| --- | --- | --- | --- | --- |
| Negative | .92 | .55 | .69 | 20 |
| Neutral | .89 | .93 | .91 | 58 |
| Positive | .70 | .95 | .86 | 22 |
| Accuracy |  |  | .86 | 100 |
| Macro average | .86 | .81 | .82 | 100 |
| Weighted average | .87 | .86 | .85 | 100 |

*Note:* Support indicates the number of statements that were incorporated.

**Figure S1**

*Care reports: Confusion Matrix of Sentiment Analysis Model*


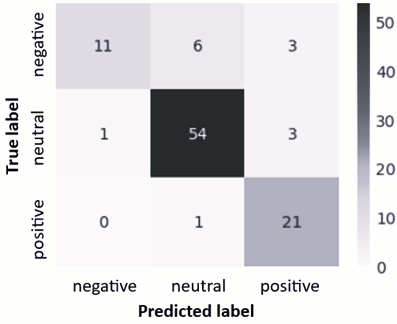


1. **Running the model on the large dataset**

We removed the manually coded reports and ran the model on the large dataset containing 574,348 unique care reports. We extracted the care reports that included predicted positive and negative sentiments. These were aggregated per week for use in the time-series analysis.

**Supporting Information B: Panel Surveys**

**Inclusion information**

Whether the person with intellectual disability lived in a sheltered home care facility was indicated by the following question: “From whom or which organization does your relative receive care, support or guidance?”. We included responses indicating care organizations for people with intellectual disabilities. In 2023, this question was removed from the survey. Therefore, for 2023 we included those individuals who received care from a care organization for people with intellectual disabilities in 2021 or 2022. Four survey responses that were only gathered in 2023 were excluded.

**Figure S2**

*Panel Surveys: Flow chart*


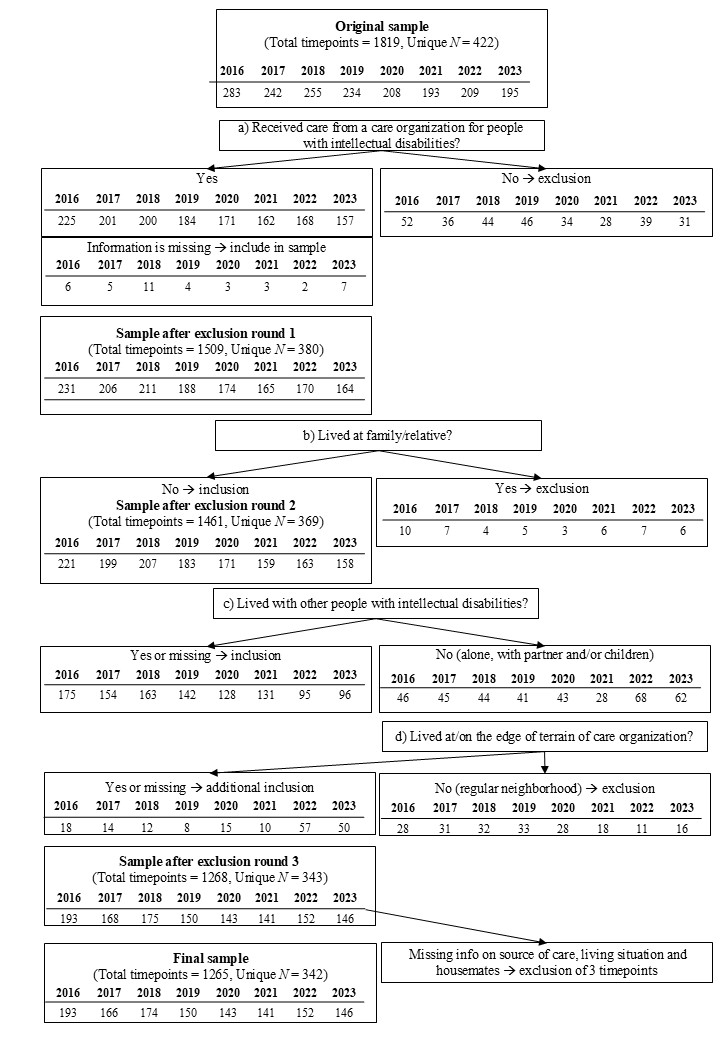


**Table S3**

*Panel Surveys: Living Situation and Living Partners of People with Intellectual Disabilities as Reported by Their Relatives*

|  | **2016** | **2017** | **2018** | **2019** | **2020** | **2021** |  | **2022** | **2023** |
| --- | --- | --- | --- | --- | --- | --- | --- | --- | --- |
| Living situation^1^  In a regular neighborhood,  not close to care organization  At care organization terrain  Close to/at the edge of a care  organization terrain  Different | 57.5%  24.4%  4.1%  8.3% | 47.6%  27.1%  7.2%  13.3% | 48.3%  35.1%  5.2%  9.8% | 50%  28.7%  6.7%  10.7% | 46.2%  31.5%  6.3%  13.3% | 47.5%  30.5%  6.4%  13.5% | Living situation  At care organization  At small-scale living organization  (e.g., living initiative, parent initiative)    Different | 96.1%^2^  -  2.6% | 67.8%  24%  6.2% |
|  |  |  |  |  |  |  |  |  |  |
| Living partners^3^  With people with intellectual  disabilities  Alone  With partner  With children (not partner)  Different | 79.3%  8.3%  1%  0%  7.8% | 82.5%  6.6%  1,2%  0.6%  7.2% | 82.2%  6.3%  0%  0.6%  9.2% | 90%  5.3%  0%  0%  2% | 84.6%  9.8%  0%  0.7%  3.5% | 87.9%  7.1%  0%  0%  3.5% | Living partners^3^  With people with intellectual disabilities  Alone, with care/common space  Alone, independently  With partner (and children)  Different | 61.2%  31.6%  5.3%  0.7%  1.3% | 61.6%  32.9%  0%  1.4%  4.1% |

^1^ Living situation had the following numbers of missing values : 11 in 2016, 8 in 2017, 3 in 2018, 6 in 2019, 4 in 2020, 3 in 2021, 2 in 2022, 3 in 2023

^2^  In 2022, the options ‘At a care institution’ and ‘At small-scale living organization (e.g., living initiative, parent initiative) were combined into: ‘At a care institution, living initiative or parent initiative’

^3^ Living partners had the following numbers of missing values: 7 in 2016, 3 in 2017 and 2018, 4 in 2019, 2 in 2020 and 2021

**Supporting Information C: additional results**

**Table S4**

*Care Reports: Weekly Aggregated Reports About Digital Contact*

|  | Reports about digital contact  (neutral, positive and negative) | | | Positive reports | | | Negative reports | | | Ratio positive/negative reports | |
| --- | --- | --- | --- | --- | --- | --- | --- | --- | --- | --- | --- |
|  | Total reports^a^ | *M* (*SD*) | Range | Total reports^a^ | *M* (*SD*) | Range | Total reports^a^ | *M* (*SD*) | Range | *M* (*SD*) | Range |
| Total sample | 844,769 | 2016.16 (587.64) | 373-3,308 | 196,796 | 469.68 (174.2) | 118-1213 | 184,224 | 439.68 (112.07) | 82-682 | 1.05 (0.19) |  |
| Severity of intellectual disability |  |  |  |  |  |  |  |  |  |  |  |
| Borderline intellectual functioning | 21,593 | 51.53 (22.43) | 2-104 | 4,071 | 9.72 (5.13) | 0-26 | 6,219 | 14.84 (7.24) | 0-35 | 0.72 (0.43) | 0-4 |
| Mild intellectual disability | 161,160 | 384.63 (117.77) | 67-598 | 37,189 | 88.76 (32.09) | 15-168 | 41,010 | 97.88 (28.33) | 19-164 | 0.9 (0.18) | 0.52-1.51 |
| Moderate intellectual disability | 113,143 | 270.03 (79.33) | 55-469 | 30,348 | 72.43 (28.43) | 20-195 | 23,657 | 56.46 (14.84) | 8-99 | 1.28 (0.34) | 0.4-3.25 |
| Severe intellectual disability | 47,204 | 112.66 (37.96) | 33-257 | 11,108 | 26.51 (15.49) | 5-127 | 5,420 | 12.94 (5.28) | 3-35 | 2.18 (1.1) | 0.56-9.33 |
| Profound intellectual disability | 17,965 | 42.88 (12.22) | 8-96 | 3,324 | 7.93 (4.06) | 0-29 | 1,233 | 2.94 (1.81) | 0-9 | 3.39 (2.67) | 0.22-16 |
| Support needs |  |  |  |  |  |  |  |  |  |  |  |
| Support without behavioural regulation | 123,047 | 293.67 (107.09) | 34-505 | 24,803 | 59.2 (25.5) | 10-163 | 24,773 | 59.12 (20.86) | 6-112 | 1.01 (0.28) | 0.5-2.31 |
| Support with behavioural regulation | 324,946 | 775.53 (202.19) | 149-1,239 | 80,017 | 190.97 (64.88) | 50-456 | 78,762 | 187.98 (42.75) | 40-282 | 1 (0.18) | 0.65-1.88 |
| Support with full care and housing | 35,711 | 85.23 (30.39) | 25-166 | 5,936 | 14.17 (8.85) | 2-63 | 3,150 | 7.52 (4.05) | 0-26 | 2.16 (1.29) | 0.3-11 |

*Note.* total reports = the sum of reports from all groups over all 419 weeks, from 2016 to 2023

^a^  The total reports include duplicates and are not equal to the sum of the subgroups as clients can appear in both severity of ID and support needs subgroups

**Post hoc analysis: Square-root transformation of time after start of the COVID-19 restrictions**

As we observed a non-linear trend in time after the start of the COVID-19 restrictions (see manuscript Figure 5 and 6), we added a square-root transformation of time to the part of Model 3 after the start of COVID-19 restrictions for reports of digital contact and the ratio of positive/negative sentiments as a post-hoc analysis (not pre-registered). For the trend over time in reports about digital contact, the inverse square-root transformation of time after the COVID-19 restrictions was statistically significant (*b* = 44.12, *SE* = 8.10, *p* < .001; see Figure S3). This pattern was also observed for the ratio of positive to negative sentiments about digital contact (*b* = 127.20, *SE* = 14.42, *p* < .001; see Figure S4). These results indicate a non-linear association between time and both outcomes in the period after the COVID restrictions were implemented. Specifically, the effect of time on digital contact reports and sentiment ratios was strongest immediately after the introduction of the restrictions, and gradually diminished over time.

**Figure S3**

*Care Reports: Inverse Square-Root Transformation of Time After the COVID-19 Restrictions*


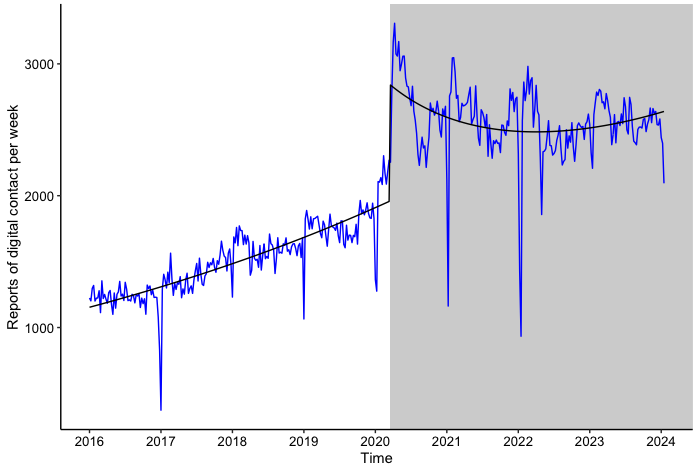


*Note*. The regression line represents the predicted values by the generalised linear quasi-poisson regression model (effect of the COVID-19 restrictions over time (*intercept* + *time***start of COVID-19 measures*), with an inverse square-root transformation of time added after the COVID-19 restrictions).

**Figure S4**

*Care Reports: Trend Over Time in the Ratio of Positive vs Negative Sentiments About Digital Contact: Inverse Square-Root Transformation of Time After the COVID-19 Restrictions*


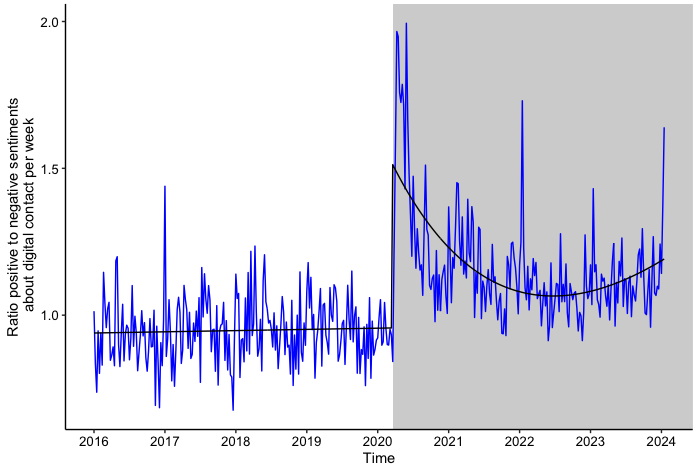


*Note*. The regression line represents the predicted values by the generalised linear quasi-poisson regression model (effect of the COVID-19 restrictions over time (*intercept* + *time***start of COVID-19 measures*), with an inverse square-root transformation of time added after the COVID-19 restrictions).

**Panel surveys: Unconditional means models**

To test whether multilevel modelling was suited for these data, we calculated intraclass correlations *(*ICCs) for the unconditional means (intercept-only) models for face-to-face contact and digital contact. For face-to-face contact, the ICC was .88 and for digital contact the ICC was .92. This indicated that 88% and 92% of the variance in face-to-face contact and digital contact respectively can be attributed to differences between participants, suggesting that a multilevel approach was suitable.

**References**

de Vries, W., van Cranenburgh, A., Bisazza, A., Caselli, T., van Noord, G., & Nissim, M. (2019). *BERTje: A Dutch BERT Model*. https://github.com/wietsedv/bertje

Menger, V., Scheepers, F., van Wijk, L. M., & Spruit, M. (2018). DEDUCE: A pattern matching method for automatic de-identification of Dutch medical text. *Telematics and Informatics*, *35*(4), 727–736. https://doi.org/10.1016/J.TELE.2017.08.002

Rutte, M. (2022). *Dutch sentiment analysis on Twitter* (Doctoral dissertation, Vrije Universiteit Amsterdam).
